# Vulnerability of wives of Nepalese labor migrants to HIV infection: a socio-epidemiological study

**DOI:** 10.1186/2049-3258-73-S1-P14

**Published:** 2015-09-17

**Authors:** Subash Thapa, Nirmala Bista, Karin Hannes, Anne Buve, Mieke Vermandere, Catharina Mathei

**Affiliations:** 1 KU Leuven, Leuven, Belgium; 2 Department of Public Health, Nobel College Pokhara University, Kathmandu, Nepal; 3 Faculty of Psychology and Educational Sciences, KU Leuven, Leuven, Belgium; 4 Department of Public Health, Institute of Tropical Medicine, Antwerp, Belgium; 5 Department of Public Health and Primary care, KU Leuven, Leuven, Belgium

## 

The vulnerability paradigm accounts for women's susceptibility to HIV infection being a consequence of socio-economic and cultural factors, and there is a strong need for socio-epidemiological analysis to understand and address vulnerability of Nepalese women to HIV infection. Therefore, to assess the risk factors and vulnerability of the wives of Nepalese labor migrants to HIV infection, we conducted a mixed-methods study in which a descriptive case study was embedded within a case-control study. A total of 224 wives of labor migrants were interviewed in the case-control study and two focus group discussions were conducted in the descriptive case study. Data was analyzed using hierarchical conditional logistic regression analysis in the case-control study and thematic analysis in the descriptive case study. We found that illiteracy, low socio-economic status and gender inequality contributed to poor knowledge and poor sexual negotiation among the wives of labor migrants and increased their risk of HIV through unprotected sex. Among male labor migrants, illiteracy, low socio-economic status, migration to India before marriage and alcohol consumption contributed to visit female sex workers and increased the risk of HIV in their wives through unprotected sex. Both labor migrants and their wives feared disclosure of positive HIV status due to HIV stigma and thus were less likely to be tested for HIV. Interventions targeting the general population, such as access to basic education, income generation and mass awareness, and interventions targeting specific subpopulations, such as gender-related training and involving men in HIV-related programs, should be combined to reduce vulnerability of Nepalese women to HIV infection.

